# Urine collection in cervical cancer screening – analytical comparison of two HPV DNA assays

**DOI:** 10.1186/s12879-020-05663-7

**Published:** 2020-12-04

**Authors:** Mette Tranberg, Jørgen Skov Jensen, Bodil Hammer Bech, Berit Andersen

**Affiliations:** 1 Department of Public Health Programmes, Randers Regional Hospital, Østervangsvej 48, 8930 Randers, NØ Denmark; 2 Department of Clinical Medicine, Aarhus University, Palle Juul-Jensens Boulevard 82, 8200 Aarhus N, Denmark; 3 Denmark Research Unit for Reproductive Microbiology, DR, Artillerivej 5, 2300 Copenhagen S, Denmark; 4 Research Unit for Epidemiology, Department of Public Health, Aarhus University, Bartholins Allé 2, 8000 Aarhus C, Denmark

**Keywords:** Self-sampling, Human papillomavirus testing, Cervical cancer screening, Urinary sample, Acceptability

## Abstract

**Background:**

To reach non-participants, reluctant to undergo clinician-based cervical cancer screening and vaginal self-sampling, urine collection for high-risk human papillomavirus detection (hrHPV) may be valuable. Using two hrHPV DNA assays, we evaluated the concordance of hrHPV positivity in urine samples in comparison with vaginal self-samples and cervical cytology samples taken by the general practitioner (GP). We also studied women’s acceptance of urine collection and preferences towards the different sampling procedures.

**Methods:**

One hundred fifty paired self-collected urine and vaginal samples and GP-collected cervical cytology samples were obtained from 30 to 59-year-old women diagnosed with ASC-US within the Danish cervical cancer screening program. After undergoing cervical cytology at the GP, the women collected first-void urine and vaginal samples at home and completed a questionnaire. Each sample was hrHPV DNA tested by the GENOMICA CLART® and COBAS® 4800 assays. Concordance in hrHPV detection between sample types was determined using Kappa (*k*) statistics. Sensitivity and specificity of hrHPV detection in urine was calculated using cervical sampling as reference.

**Results:**

With the COBAS assay, urine showed good concordance to the vaginal (*k* = 0.66) self-samples and cervical samples (*k* = 0.66) for hrHPV detection. The corresponding concordance was moderate (*k* = 0.59 and *k* = 0.47) using CLART. Compared to cervical sampling, urinary hrHPV detection had a sensitivity of 63.9% and a specificity of 96.5% using COBAS; compared with 51.6 and 92.4% for CLART. Invalid hrHPV test rates were 1.8% for COBAS and 26.9% for CLART. Urine collection was well-accepted and 42.3% of the women ranked it as the most preferred future screening procedure.

**Conclusions:**

Urine collection provides a well-accepted screening option. With COBAS, higher concordance between urine and vaginal self-sampling and cervical sampling for hrHPV detection was found compared to CLART. Urinary hrHPV detection with COBAS is feasible, but its accuracy may need to be improved before urine collection at home can be offered to non-participants reluctant to both cervical sampling and vaginal self-sampling.

## Background

Compared with cytology-based screening, high-risk human papillomavirus (hrHPV) DNA testing has superior clinical sensitivity for detecting Cervical Intraepithelial Neoplasia grade 3 or worse (CIN3+) and improved negative predictive value [1, 2]. Another advantage of hrHPV testing is that, unlike cytology, it allows non-participating women to self-sample a vaginal sample in their own home using a device and return the sample to the laboratory for hrHPV testing by mail [3]. Home-based vaginal self-sampling is a well-accepted screening method, proven to increase screening participation significantly compared to mailed reminders to attend clinician-based screening [3, 4]. Nevertheless, even with the vaginal self-sampling offer, some women are still not engaged in screening, possibly explained by the woman’s uncertainty about proper self-sampling including fear of injuries and discomfort touching herself [5]. Urine samples may be considered as an alternative self-sampling screening option as it is cheap, non-invasive and straightforward to collect [6–8].

Several studies have assessed the hrHPV concordance and agreement between urine samples versus vaginal self-samples and clinician-collected cervical samples using a great variety of hrHPV DNA assays [9–15]. However, little attention has been given to the direct comparison of different hrHPV DNA assays within the same study [12, 15]; thus no strong conclusions about assay effects can be made. Moreover, the performance of hrHPV testing on urine samples has mainly been evaluated in studies, where women obtained the urine samples in the clinic and the samples were transported to the laboratory immediately for analyses [9–13, 15]. Yet, if urine collection is to be implemented in an organized screening program, a home-based setting with mailing of the samples to the laboratory would be desirable. Urine collection at clinics has been reported to be highly acceptable [10, 13] and preferable over vaginal self-sampling and clinician-based screening [16, 17]; yet data regarding women’s acceptability and preferences towards home-based urine collection are lacking.

### Aims

We evaluated the concordance of hrHPV positivity in urine samples collected at home in comparison with vaginal self-samples collected at home and cervical cytology samples collected by a general practitioner (GP) using two hrHPV DNA assays (COBAS® 4800 and GENOMICA CLART® HPV4S). We also assessed the women’s acceptance of urine collection and preferences towards the different sampling procedures.

## Methods

### Setting

In Denmark, cervical cancer screening is a nationwide program inviting women aged 23–64 years for liquid-based cervical cytology sampling at their GP (cervical sample) [18]. At present, women aged 23–59 years are screened with cytology, whereas women aged 60–64 years undergo hrHPV-based screening [18]. Women aged 30–59 years who are diagnosed with atypical squamous cells of undetermined significance (ASC-US) undergo reflex hrHPV triage testing, and ASC-US/hrHPV positive women are referred for colposcopy, whereas ASC-US/hrHPV negative women are referred back to the routine screening program [18]. This study was conducted in the Central Denmark Region (CDR), where all cervical cytologies are routinely handled and analyzed by the Department of Pathology, Randers Regional Hospital. In the CDR, the COBAS**®** 4800 (Roche Diagnostics, Switzerland) test is the routine test platform for hrHPV DNA analysis.

### Study participants

Women eligible for this study were aged 30–59 years and diagnosed with ASC-US based on a cervical sample between June 2015 and December 2016. Exclusion criteria were pregnancy and having given birth within the last 3 months. The recruitment procedure has been described in detail in a previous publication assessing the hrHPV concordance between home-based vaginal self-sampling and cervical sampling when using COBAS for analysis [19]. In brief, eligible women were consecutively mailed a consent form and an information letter about the study explaining that they had to contact the investigator for oral information regarding the study and return a signed informed consent if they wanted to participate.

### Sample collection, processing and storage

As per routine, the women had a cervical sample collected with a cervical cytobrush (Cervex-Brush® Combi, Rovers Medical Devices, B. V, Oss, Netherlands) at their GP [20]. The Cervex-Brush device enables simultaneous collection of cells from the ectocervix, the endocervix and the transformation-zone in a single sample [20]. After collecting cervical epithelial cells, the cervical cytobrush was rinsed in 10 mL SurePath medium (BD Diagnostics, Burlington, NC) and mailed to the Department of Pathology, Randers Regional Hospital, for routine processing and testing as previously described [19]. The COBAS hrHPV testing was performed as per routine using the sample cell pellet from 1 mL SurePath medium. For this study, 100 μl of the residual purified DNA material was subsequently stored at -80 °C prior to CLART testing.

After undergoing cervical sampling at the GP and written informed consent was obtained, the women were mailed a self-sampling package to their home. The package included a dry brush device (Evalyn**®** Brush, Rovers Medical Devices, B. V, Oss, Netherlands) for vaginal self-sampling [21], a transportation tube with preservative media (Genelock, ASSAY ASSURE, Sierra Molecular, CA) for urine sampling, written and picture-based instructions showing the order of the self-sampling, pre-addressed return envelope, and a questionnaire. The women were further asked to firstly collect the vaginal sample and secondly collect a first-void urine sample (the first of 10–12 mL of urine voided) in a plastic cup during their first urination in the morning. The urine was transferred to the provided transportation tube by the participant. The women were urged to collect both samples and return the samples and the accompanying questionnaire by ordinary mail on the same day as the samples were taken and before an eventual colposcopy examination [19].

Upon arrival in the laboratory, the urine sample was stored overnight at 4 °C and then vortexed for 5 min. A 10–12 mL volume of urine was centrifuged at 3000 x RPM for 20 min at room temperature. After centrifugation, the cell pellet was re-suspended in 1 mL 25% ethanol-buffered (TRIS) and stored at -80 °C until further hrHPV testing. The dry brush head was transferred into 10 mL SurePath medium (BD Diagnostics, Burlington, NC) also stored overnight at 4 °C and then vortexed for 5 min. A 6.4 mL volume of the self-sample material was centrifuged at 3000 x RPM for 20 min at room temperature [19]. With the supernatant removed, the cell pellet was placed in 1 mL 25% ethanol-buffered (TRIS) and stored at -80 °C until further hrHPV testing [19]. A volume of 6.4 mL was chosen to correct for the material volume used for cytology examination performed on the cervical sample [19]. The median time interval between collecting the self-samples at home and the samples being processed and stored at -80 °C at the laboratory was 3 days (range: 2 to 8 days).

### HrHPV DNA testing

Before the day of COBAS hrHPV DNA testing, the cell pellet material from the urine and vaginal self-samples were thawed overnight at 4 °C (after storage for 4 days to 18 months, median: 10 months). Subsequently, the self-samples (1 mL volume) were vortexed for 15 s before being transferred to empty test tubes for DNA purification and hrHPV testing [19]. DNA was purified using the COBAS × 480, and amplification and detection of hrHPV DNA were undertaken using the COBAS z480 analyzer [22]. From each self-sample, 100 μl of the residual purified DNA material were stored at -80 °C, until further CLART hrHPV testing. The COBAS**®** assay is a fully automated real-time PCR-based method, separately detecting HPV16, HPV18, and 12 other hrHPV types (HPV31, 33, 35, 39, 45, 51, 52, 56, 58, 59, 66 and 68) including the beta-globin gene as an extraction and amplification control [22].

Before the day of the CLART hrHPV DNA testing (HPV4S, GENOMICA, Madrid, Spain), the residual purified DNA material from the self-samples and the cervical samples gained using COBAS × 480, were thawed overnight at 4 °C (after storage for 2 to 25 months, median: 12 months). Five μl of the purified DNA material from the samples were used for the PCR amplification. The PCR amplification was performed using the CLART HPV4S amplification kit (GENOMICA) [23]. Detection was performed on the CLART microarray. The genotyping results were analyzed and automatically performed on the Clinical Array Reader (GENOMICA) [23]. CLART HPV4S detects 14 hrHPV genotypes individually (HPV16, 18, 31, 33, 35, 39, 45, 51, 52, 56, 58, 59, 66, and 68) and two low-risk HPV genotypes (6 and 11). Amplification of a spiked CFTR plasmid served as an internal control of the PCR process, while the internal control for human CFTR gene validates specimen quality in the sample [23].

For both assays, samples with an invalid test result (i.e. COBAS: no betaglobin gene detected, CLART: no human CFTR amplification detected, or no spiked CFTR plasmid amplification detected) were retested once on diluted samples, and the second result was considered definitive. To avoid between-run discrepancy, the self-samples belonging to the same woman were processed in the same run for the COBAS analysis; and similarly for the CLART analysis the self-samples and the cervical sample belonging to the same woman were processed in the same run. Every run included four water samples to measure contamination [19]. The laboratory personnel performed the hrHPV testing without knowledge of the hrHPV status of the cervical samples [19].

### HrHPV positivity and histological results

Any hrHPV positivity was defined as samples being positive for HPV16, 18, 31, 33, 35, 39, 45, 51, 52, 56, 58, 59, 66, or 68, whereas positivity for specific hrHPV genotypes were grouped into “HPV16/18” and “hrHPV other” including HPV31, 33, 35, 39, 45, 51, 52, 56, 58, 59, 66, and 68. If the sample was positive for low-risk HPV types only (HPV6 and 11), it was classified as hrHPV negative. As per routine, histological results were only available for women with an hrHPV positive cervical sample [19]. The results were classified following the CIN classification and grouped into normal tissue, CIN (not specified), CIN1, and CIN2+ (including CIN2, CIN3/AIS, and carcinoma). The most severe histological result was used if more were available. Test results of the cervical samples and the histological samples were obtained from the nationwide Danish Pathology Data Bank [24], while test results of the self-samples were retrieved from the Department of Pathology.

### Acceptability and preferences

Together with the self-sampling package, the women received a questionnaire (see supplementary material) addressing among others the acceptance of urine collection, the clarity of the user instructions, and the confidence in correct execution of the urine collection. For analysis, the five response categories were grouped into three: “Agree” (totally agree and agree), “Disagree” (disagree or totally disagree), and “Do not know”. Women were also requested to rank if they preferred urine collection, vaginal self-sampling, or cervical sampling at the GP as their future screening examination. For analysis, women who refrained to rank their preferences or ranked two or more sampling methods as their preferred method were coded as missing. Additionally, the women were asked to report the date of collecting their self-sample and whether they had engaged in sexual intercourse between the cervical sampling at the GP and self-sampling [19].

### Statistical analyses

For analysis, only women with valid hrHPV results from paired urine, vaginal, and cervical samples for both hrHPV assays were included. In the following, the term concordance refers to kappa values, while the term agreement corresponds to percentage agreement. Cohen’s kappa (κ) was used to measure concordance in hrHPV positivity (any hrHPV and specific genotypes) for urine vs vaginal self-samples, urine vs cervical samples by the COBAS and CLART assays. Concordance was defined as “poor” (κ ≤ 0.20), “fair” (0.21 ≤ κ ≤ 0.40), “moderate” (0.41 ≤ κ ≤ 0.60), “good” (0.61 ≤ κ ≤ 0.80), or “very good” (κ ≥ 0.81) [25]. Concordance estimates were presented with 95% confidence intervals (CIs). Comparing the presence of HPV16/18 and hrHPV other types between the samples; concordance was determined by the presence of at least one identical genotype in both samples (i.e. HPV16/18 only, HPV16/18 and other types, and other types only); discordance was determined as no genotype similarities [19]. McNemars test was performed to compare proportions of hrHPV positive results between the paired sample types. We also calculated the overall percentage of agreement between the paired samples including 95% CIs [19]. Stratified by hrHPV assay, the sensitivity and specificity of hrHPV positivity (any hrHPV and specific genotypes) in urine samples was calculated with 95%CIs using the vaginal self-sample or the cervical sample as comparator test.

The acceptability of urine collection and preferences regarding the different sampling procedures were evaluated by descriptive statistics (proportions and 95% CIs). We tested whether women’s preferences differed between age groups (30–39, 40–49 and 50–59 years). The χ2-test was used to test for differences in categorical data. For continuous data, medians and interquartile ranges (IQR) were calculated; the Mann Whitney rank sum test was used to test for differences. *P*-values < 0.05 were considered statistically significant. The statistical analyses were performed using STATA, version 16 (STATA College). A sample size calculation has been reported elsewhere [19].

### Ethical approval

According to the EUs General Data Protection Regulation, the project was listed at the record of processing activities for research projects in the CDR (j.no.:1–10–72-69-15). The study was approved by the local Ethical Committee of the CDR (j.no.:1–16–02-209-15).

## Results

### Study population

**Fig. 1:**
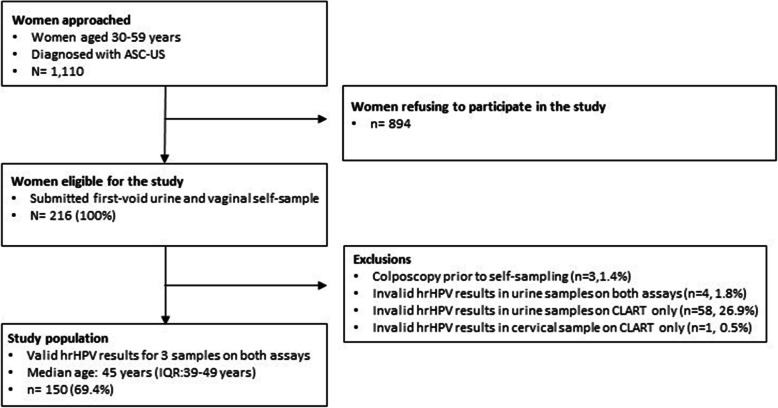
Flowchart depicting the study population. Figure notes: ASC-US: Atypical Squamous Cells of Undetermined Significance, IQR: InterQuartile Range. hrHPV: High Risk Human PapillomaVirus

Of the 1110 eligible women, 216 participated in the study by returning urine and vaginal self-samples. However, a total of 66 women (30.6%) were excluded from the analyses: Three women (1.4%) had colposcopy performed prior to self-sampling; four women (1.8%) had invalid hrHPV urine results on both assays; 58 women (26.9%) had invalid hrHPV urine results using CLART only; and one woman (0.5%) had invalid cervical hrHPV result using CLART only (Fig. 1). The remaining 150 women (69.4%) constituted the study population. Median age of women in the study population was 45 years (IQR: 39 to 49 years). The cervical samples and self-samples were collected with a median time interval of 42 days (IQR: 33 to 52 days). Histological results were available for 36 women; of whom 11 (30.6%), 4 (11.0%), 10 (27.8%), and 11 (30.6%) received a diagnosis of normal tissue, CIN (not specified), CIN1, and CIN2+, respectively.

### HrHPV positivity in urine, vaginal and cervical samples

For both assays, the hrHPV positivity was statistically significantly lower in urine samples compared to vaginal self-samples except for HPV16/18 positivity using CLART (Table 1). The hrHPV positivity in urine samples was also lower compared to cervical samples for both assays, but in this comparison only HPV16/18 positivity using COBAS was statistically significant (4.0% vs 10.0%, *p* = 0.02) (Table 1). No statistically significant differences were observed in hrHPV positivity (any and specific genotypes) between the COBAS and CLART assay for the urine samples (*p* > 0.05 for all comparisons, data not shown).

**Table 1:** hrHPV positivity (any and specific genotypes) for urine, vaginal and cervical samples

hrHPV result	Assay (total)	Urine positive n, % (95%CI)		Vaginal positive n, % (95%CI)		Cervical positive n, % (95%CI)		McNemar test Urine vs. vaginal	McNemar test Urine vs. cervical
**Any hrHPV (14 types)**^**a**^	**Cobas** (*n* = 150)	27	18.0 (12.2–25.1)	44	29.3 (22.2–37.3)	36	24.0 (17.4–31.6)	0.01	0.05
	**Clart** (n = 150)	25	16.7 (11.1–23.6)	35	23.3 (16.8–30.9)	31	20.7 (14.5–28.0)	0.04	0.31
**HPV16/18**^**b,d**^	**Cobas** (n = 150)	6	4.0 (1.5–8.5)	17	11.3 (6.7–17.5)	15	10.0 (5.7–15.9)	0.01	0.02
	**Clart** (n = 150)	4	2.7 (0.7–6.7)	9	6.0 (2.8–11.1)	8	5.3 (2.3–10.2)	0.06	0.13
**hrHPV other (12 types)**^**c**,**d**^	**Cobas** (n = 150)	24	16.0 (10.5–22.9)	38	25.3 (18.6–33.1)	29	19.3 (13.3–26.6)	0.01	0.27
	**Clart** (n = 150)	21	14.0 (8.9–20.6)	31	20.7 (14.5–28.0)	29	19.3 (13.3–26.6)	0.04	0.17

^a^ Any hrHPV: HPV16 and/or HPV18 and/or HPV31, 33, 35, 39, 45, 51, 52, 56, 58, 59, 66 and 68

^b^ HPV16/18: HPV16 and/or HPV18 including co-infections with hrHPV of other types

^c^ HrHPV other: HPV31, 33, 35, 39, 45, 51, 52, 56, 58, 59, 66 and 68 including co-infections with HPV16/18

McNemars test comparing the hrHPV positivity proportions between sample types

^d^ Women with co-infections with HPV16/18 and hrHPV of other types appear in both sub-analyses

^%^ Row percentage

### Concordance between urine and vaginal self-sampling

Using COBAS for analysis, the concordance between the urine and vaginal self-samples was good for any hrHPV (κ =0.66 and agreement: 87.3%) and hrHPV other types (κ =0.68 and agreement: 89.3%), while moderate concordance between samples was found for HPV16/18 (κ =0.49, agreement: 92.7%) (Table 2). When CLART was used, the hrHPV concordance was moderate with κ values ranging from 0.54 to 0.60 with the highest agreement seen for HPV16/18 (96.7%). The sensitivity and specificity of any hrHPV positivity in urine using vaginal samples as reference was insignificantly higher using COBAS as compared with CLART (59.1% (95% CI: 43.2–73.7%) and 99.1% (95% CI: 94.9–99.7%) vs 57.1% (95% CI:39.4–73.7%) and 95.7% (95% CI: 90.1–98.6%)) (*p* = 0.87 and 0.12) (Table 2). Among the 11 CIN2+ cases, a total of six CIN2+ cases had hrHPV negative urine results; out of which two cases were tested negative on both assays and four cases were tested negative on CLART only. None of the six cases reported problems in collecting the urine sample. With respect to vaginal sampling, all 11 CIN2+ cases were tested hrHPV positive using COBAS, while one case was missed by the CLART assay.

**Table 2:** Urine versus vaginal self-samples: Concordance and agreement for hrHPV positivity and analytical accuracy of urine using vaginal self-samples as comparator test

hrHPV result	Assay (total)		Vaginal positive	Vaginal negative	Kappa^**d**^ (95%CI)	Agreement^**e**^ (%)(95%CI)	Sensitivity (%)(95%CI)	Specificity (%)(95%CI)
**Any hrHPV**^**a**^ **(14 types)**	**Cobas** (*n* = 150)	Urine positive	26	1				
		Urine negative	18	105	0.66 (0.52–0.79)	87.3 (80.9–92.2)	59.1 (43.2–73.7)	99.1 (94.9–99.7)
	**Clart** (n = 150)	Urine positive	20	5				
		Urine negative	15	110	0.59 (0.43–0.75)	86.7 (80.2–91.7)	57.1 (39.4–73.7)	95.7 (90.1–98.6)
**HPV16/18**^**b,f**^	**Cobas** (n = 150)	Urine positive	6	0				
		Urine negative	11	133	0.49 (0.23–0.74)	92.7 (87.3–96.3)	35.3 (14.2–61.7)	100.0 (97.3–100.0)
	**Clart** (n = 150)	Urine positive	4	0				
		Urine negative	5	141	0.60 (0.29–0.92)	96.7 (92.4–98.9)	44.4 (13.7–78.8)	100 (97.4–100.0)
**hrHPV other**^**c,f**^ **(12 types)**	**Cobas** (n = 150)	Urine positive	23	1				
		Urine negative	15	111	0.68 (0.54–0.82)	89.3 (83.3–93.8)	60.5 (43.4–75.9)	99.1 (95.1–99.9)
	**Clart** (n = 150)	Urine positive	16	5				
		Urine negative	15	114	0.54 (0.36–0.71)	86.7 (80.2–91.7)	51.6 (33.1–69.8)	95.8 (90.5–98.6)

^a^ Any hrHPV: HPV16 and/or HPV18 and/or HPV31, 33, 35, 39, 45, 51, 52, 56, 58, 59, 66 and 68

^b^ HPV16/18: HPV16 and/or HPV18 including co-infections with hrHPV of other types

^c^ HrHPV other: HPV31, 33, 35, 39, 45, 51, 52, 56, 58, 59, 66 and 68 including co-infections with HPV16/18

^d^ Cohens Kappa. “Poor” (κ ≤ 0.20), “fair” (0.21 ≤ κ ≤ 0.40), “moderate” (0.41 ≤ κ ≤ 0.60), “good” (0.61 ≤ κ ≤ 0.80), or “very good” (κ ≥ 0.81) [25]

^e^ Percentage of all samples that give concordant results

^f^ Women with co-infections with HPV16/18 and hrHPV of other types appear in both sub-analyses

### Concordance between urine and cervical samples

Using COBAS for analysis, the concordance between urine and cervical samples was good (κ values ranging from 0.66 to 0.70 and agreement from 88.7 to 91.3%), except for the detection of HPV16/18 where a fair concordance was found (κ: 0.34 and agreement: 91.3%) (Table 3). Using CLART for analysis, concordance was moderate for any hrHPV (κ =0.47), good for HPV16/18 (κ =0.65), and fair for hrHPV other types (κ =0.38) with agreement ranging from 82.7 to 97.3%. Using the cervical sample as reference, the sensitivity and specificity of any hrHPV detection in urine samples was insignificantly higher using COBAS as compared with CLART (63.9% (95% CI: 46.2–79.2%) and 96.5% (95% CI: 91.3–99.0%) vs 51.6% (95% CI: 33.0–69.8%) and 92.4% (95% CI: 86.1–96.5%)) (*p* = 0.30 and 0.17) (Table 3). Using COBAS, a total of 17 discordant pairs (11.3%) occurred. The median number of days between the samples was significantly higher for the 13 cervical sample hrHPV positive/urine hrHPV negative women as compared with the 23 women having positive concordant results (44 vs 33 days, *p* = 0.03). Four women were GP-sample hrHPV negative/urine hrHPV positive; out of whom one participant reported having no sexual intercourse in the time interval between the samples.

**Table 3:** Urine versus cervical-samples: Concordance and agreement for hrHPV positivity and analytical accuracy of urine using cervical samples as comparator test

hrHPV result	Assay (total)		Cervical positive	Cervical negative	Kappa^**d**^ (95%CI)	Agreement^**e**^ (%)(95%CI)	Sensitivity (%)(95%CI)	Specificity (%)(95%CI)
**Any hrHPV**^**a**^ **(14 types)**	**Cobas** (n = 150)	Urine positive	23	4				
		Urine negative	13	110	0.66 (0.51–0.81)	88.7 (82.5–93.3)	63.9 (46.2–79.2)	96.5 (91.3–99.0)
	**Clart** (n = 150)	Urine positive	16	9				
		Urine negative	15	110	0.47 (0.30–0.65)	84.0 (77.1–89.5)	51.6 (33.0–69.8)	92.4 (86.1–96.5)
**HPV16/18**^**b,f**^	**Cobas** (n = 150)	Urine positive	4	2				
		Urine negative	11	133	0.34 (0.08–0.61)	91.3 (85.6–95.3)	26.7 (0.07–55.1)	98.5 (94.8–99.8)
	**Clart** (n = 150)	Urine positive	4	0				
		Urine negative	4	142	0.65 (0.34–0.97)	97.3 (93.3–99.3)	50.0 (15.7–84.3)	100.0 (97.4–100.0)
**hrHPV other**^**c**,**f**^ **(12 types)**	**Cobas** (n = 150)	Urine positive	20	4				
		Urine negative	9	117	0.70 (0.55–0.85)	91.3 (85.6–95.3)	69.0 (49.2–84.7)	96.7 (91.8–99.1)
	**Clart** (n = 150)	Urine positive	12	9				
		Urine negative	17	112	0.38 (0.19–0.57)	82.7 (75.6–88.4)	41.4 (23.5–61.1)	92.6 (86.3–96.5)

^a ^Any hrHPV: HPV16 and/or HPV18 and/or HPV31, 33, 35, 39, 45, 51, 52, 56, 58, 59, 66 and 68

^b^ HPV16/18: HPV16 and/or HPV18 including co-infections with hrHPV of other types

^c^ HrHPV other: HPV31, 33, 35, 39, 45, 51, 52, 56, 58, 59, 66 and 68 including co-infections with HPV16/18

^d ^Cohens Kappa. “Poor” (κ ≤ 0.20), “fair” (0.21 ≤ κ ≤ 0.40), “moderate” (0.41 ≤ κ ≤ 0.60), “good” (0.61 ≤ κ ≤ 0.80), or “very good” (κ ≥ 0.81) [25]

^e^ Percentage of all samples that give concordant results

^f ^ Women with co-infections with HPV16/18 and hrHPV of other types appear in both sub-analyses

When CLART was used, 24 discordant pairs (16.0%) were observed, out of whom 15 women were cervical sample hrHPV positive/urine hrHPV negative (Table 3). No significant difference in the median number of days between the samples was found for these 15 women compared to the 16 women with positive concordant test results (43 vs 33 days, *p* = 0.78). Nine women were cervical sample hrHPV negative/urine hrHPV positive; out of whom three women (33.3%) reported having no sexual intercourse in the time separating the samples.

### Women’s accept and preferences

A total of 149 women answered the questionnaire. Most women agreed that collection of the urine sample at home were easy (97.3%, 95% CI: 93.3–99.3%), comfortable (97.3%, 95% CI: 93.9–99.3%), and that the user instructions were clear (98.7%, 95% CI: 95.2–99.8%). Five women (3.4%, 95% CI: 1.1–7.7%) were uncertain about the proper collection of the urine samples, but none of the women provided explanations for their uncertainty. A total of 111 (74.5%) women ranked their preferences regarding the different sampling procedures. Urine sampling was the most preferred method (ranked first by 47 (42.3%, 95% CI: 33.1–52.1%)), followed by cervical sampling at the GP (ranked first by 36 (32.4%, 95% CI: 23.9–41.9%)), and vaginal self-sampling (ranked first by 28 (25.2%, 95% CI: 17.5–34.4%)). Preferences for sampling procedures did not vary significantly by age groups (*p* = 0.80).

## Discussion

### Main findings

With the COBAS assay, urine samples showed good concordance in hrHPV detection compared with vaginal and cervical samples, while moderate hrHPV concordance was found between samples using the CLART assay. Compared to cervical sampling, urinary hrHPV detection had a sensitivity of 63.9% and a specificity of 96.5% using COBAS; compared with 51.6 and 92.4% for CLART. The invalid hrHPV test rates of urine were 1.8 and 26.9% for the COBAS and CLART assay, respectively. Home-based urine collection was well-accepted and women ranked it as the most preferred future screening procedure.

### Strengths and limitations

Main strengths of our study were that the women served as their own controls, limiting potential biases and that the women collected the urine samples at home without supervision from healthcare professionals. From an implementation point-of-view, this is the most appropriate setting to evaluate urine collection before becoming routine. Additionally, we used two clinically validated hrHPV DNA assays approved for primary hrHPV screening [23, 26, 27]. Yet, the clinical validation of the CLART assay should be interpreted with some caution since it was validated against a comparator assay (MGP-PCR) that had not itself been clinically validated according to international guidelines [23]. A limitation of the study is the time interval between samples. Part of the discrepancy in hrHPV concordance between urine and cervical samples may be explained by a hrHPV infection acquired or cleared between sampling [19]. Yet, the questionnaire data enabled us to interpret discordant results, and the time interval between collecting the samples was not an issue when comparing urine samples with vaginal self-samples. Another reason for this discrepancy may stem from the recommended sampling order collecting the vaginal sample first and the first-void urine sample second. It could be speculated if this procedure may have reduced the amount of HPV containing mucus and debris from exfoliated cells from the female genital organs including the cervix which are captured with the first-void urine flow [6]. Ideally, the order of the urine and vaginal sampling should have been randomized. Another limitation is that the study was not designed to assess the clinical accuracy of urinary hrHPV detection. Therefore, further evaluation of the clinical accuracy of urine samples collected at home is warranted. A high number of women refused to participate in the study (80.5% non-participants). This may have caused selection bias if participants differed from non-participants in their ability to collect the urine sample correctly. However, because urine collection is already widely used for other purposes and is generally trusted and accepted by women this scenario is considered less likely.

Since our study was conducted in a referral population diagnosed with ASC-US, the concordance results cannot be generalized directly to a screening population. Furthermore, as we enrolled women who had already attended the screening program, the acceptability of urine collection may differ from women reluctant to participate in routine screening. A generalization of our results to routine screening programs should therefore be done with caution.

### Interpretation and comparison with previous studies

Even though first-void urine was used, which has been proven to contain significantly more HPV DNA than the subsequent part [8, 28], we found lower hrHPV positivity in urine samples as compared with corresponding cervical and vaginal samples for both assays. Our result corresponds to most comparative studies [9–12, 15, 29, 30], but not all [13, 14, 28]. In this study, a specially designed device was not used for first-void urine collection. This may have reduced the amount of HPV collected in the urine samples as supported by data showing higher HPV concentrations in urine samples collected with a specially designed device than simple urine-cup collected samples [31].

COBAS showed higher concordance for hrHPV detection (any type) between first-void urine and cervical samples than CLART (κ =0.66 vs 0.47). Our concordance using COBAS was higher than reported by Asciutto et al. (κ =0.58) [9] and Cho et al. (κ =0.33) [12], but lower than reported by Bernal et al. (κ =0.76) [14] and Sargent et al. (κ =0.82) [10]; all using a combination of first-void urine samples and COBAS in referral populations. As our study was the only one with an interval between the urine and cervical sample, some of the differences in concordance may be attributed to this factor. Other reasons for the differences are likely explained by variations in sampling procedure (at home vs clinic), type of preservative media [28], (pre-) analytical processing protocols [13], storage conditions [28], the volume of urine collected [28], and study populations (differences in abnormal cytology prevalence). The concordance between urine and cervical samples for hrHPV detection using COBAS remained robust (κ =0.61), when including the 58 women who had an invalid test result by CLART only in the analysis (data not shown).

A meta-analysis found a pooled sensitivity of 77% and specificity of 88% for hrHPV detection in urine samples compared with cervical samples [8]. In our study, lower sensitivity (63.9 and 51.6% for COBAS and CLART, respectively) but higher specificity (96.5 and 92.4% for COBAS and CLART, respectively) was found between the urine and cervical samples for both assays. However, it is possible that adjustment of the COBAS assay cut-off value for positive result could provide performance-related benefits for urine collection, as reported elsewhere [29]. Despite small numbers, CLART showed higher concordance between urine and cervical samples for the detection of HPV16/18 than COBAS (κ =0.65 vs 0.34), whereas COBAS performed better than CLART for the detection of hrHPV other types (κ =0.70 vs 0.38). In comparison with our previous study which was conducted within the same study population (*n* = 213), good concordance (κ =0.70) was found between vaginal self-sampling and cervical sampling for hrHPV detection using COBAS including sensitivity of 80.9% and specificity of 91.6% [19]. Here, data showed higher concordance for the detection of HPV16/18 than hrHPV other types (κ = 0.73 vs 0.64). Additionally, vaginal self-sampling was well-accepted, but almost 10% of the women expressed concerns about proper sampling. Here, no CIN2+ cases were missed by vaginal self-sampling [19].

In the present study, overall agreement between urine and vaginal self-samples for detection of hrHPV (any type) using COBAS (87.3%) was in line with one previous study [11].

Neither the COBAS nor the CLART assays are currently CE-marked for urinary testing; yet, urine samples analyzed on CLART resulted far more often in invalid hrHPV test results than on COBAS (26.9% vs 1.8%). This indicates that the CLART assay may be more severely affected by the presence of PCR inhibitors in urine and the lower amount of cells in urine, both known to reduce assay sensitivity [32, 33]. In comparison, invalid test rates of urine samples have in other studies been reported to range between 0 and 4% using PCR-based HPV DNA assays [9, 10, 12–15, 30]. The longer storage of the urine samples before the CLART analysis as compared with COBAS (12 vs 10 months) may have contributed to the high invalid test rates. However, there was no significantly difference in the median storage time between the 150 women having valid urine results on CLART as compared to the 58 excluded women with invalid urine results on CLART only (12 vs 13 months, *p* = 0.90). Whether optimizing the (pre-)analytical processing protocols using i) more concentrated purified DNA material and ii) the MagNA Pure 96 platform (Roche Diagnostics, Switzerland) for DNA extraction as utilized in the clinical validation study of the CLART assay [23] could lead to better results for the assay warrants further exploration. However, our findings indicate that the CLART assay may not be the preferred choice for urinary hrHPV detection. Indeed, our results support that future research should focus on optimizing the urinary (pre)-analytical procedures to improve accuracy, but also compare accuracy of hrHPV testing in paired urine and cervical samples using different combinations of urine collection methods and hrHPV assays. The ongoing VALUDES study seeks to address this current lack in evidence [34].

For screening purposes, detection of hrHPV in urine would be useful only if it can identify women who have underlying CIN2+, which is a treatable screening endpoint [35]. Clinical accuracy of hrHPV testing with COBAS for CIN2+ detection using urine samples has proven to be significantly lower than compared with cervical and vaginal sampling [29]. Although our study was not designed to evaluate the clinical accuracy of urine, we did find that up to half of the CIN2+ cases were missed by urine collection.

For use in screening, a high acceptability of the method is of great importance if wanting to reach non-participants through urine collection. Our results were consistent with the literature, showing urine collection to be highly acceptable [10, 13]. Similar to other studies, urine was the most preferred screening method [13, 16, 17]. We did not observe any differences in preferences between age groups. Confidence in correct execution of the urine collection procedure is essential, as insecurity could lead to mistrust of the test results and cause the woman to worry. Despite women in our study performed urine collection at home with no specially designed urine collection device [31], only 3.4% of the women expressed concerns about collecting the urine sample correctly. This is lower than the 20% reported by women performing clinic-based urine collection [10] and even lower than the 10% of women expressing concerns about performing home-based vaginal self-sampling as reported previously [19]. In our study, slightly more women preferred cervical sampling at the GP over vaginal self-sampling (32.4% vs 25.2%). This finding may reflect lack of confidence in one’s own ability to perform vaginal self-sampling correctly. On top of this, the enrolled women had already participated in the screening program which may have caused them to prefer cervical sampling. However, the nature of the questionnaire data does not enable us to investigate this further.

## Conclusions

This study showed that home-based urine collection was well-accepted and ranked as the most preferred future screening procedure. The COBAS assay performed better than CLART with respect to higher hrHPV concordance between urine and both vaginal self-sampling and cervical sampling as well as fewer invalid hrHPV test results. This study thus confirms the utility of urinary hrHPV detection with COBAS although its accuracy may need to be improved before urine can serve as an alternative screening option to reach non-participants reluctant to undergo GP-based cervical sampling and vaginal self-sampling.

## Supplementary Information

**Additional file 1:.** Questionnaire.

## Data Availability

The dataset used in this study are not publicly available, but an aggregated dataset might be available from the corresponding author upon reasonable request and permission from relevant Danish Authorities.

## Abbreviations

AIS: Adenocarcinoma In Situ
ASC-US: Atypical Squamous Cells of Undetermined Significance
CDR: Central Denmark Region
CI: Confidence Interval
CIN: Cervical Intraepithelial Neoplasia
CIN1: Cervical Intraepithelial Neoplasia of grade 1
CIN2+: Cervical Intraepithelial Neoplasia of grade 2 or worse
CIN3: Cervical Intraepithelial Neoplasia of grade 3 (CIN3)
GP: General Practitioner
hrHPV: High-risk Human Papilloma Virus
IQR: InterQuartile Ranges
PCR: Polymerase Chain Reaction
