# Supplementary material for: Urine collection in cervical cancer screening – analytical comparison of two HPV DNA assays
Source: BMC Infect Dis. 2020 Dec 4;20:926. doi: 10.1186/s12879-020-05663-7 (PMC7716507; doi:10.1186/s12879-020-05663-7)
Supplement: Supplementary file 1 — Additional file 1:. Questionnaire. [file 12879_2020_5663_MOESM1_ESM.docx]

# Questionnaire

1. Date for collecting the urine and cervico-vaginal self-samples.

_____________________________________

1. Have you had sexual intercourse in between the cervical sampling at the general practitioner (GP) and collecting the urine and cervico-vaginal self-samples?

□ Yes

□ No

**The following questions are about your experience with urine collection.**

1. It was easy to collect the urine sample?

| Totally agree | Agree | Disagree | Totally disagree | Do not know |
| --- | --- | --- | --- | --- |
|  |  |  |  |  |

1. It was uncomfortable to collect the urine sample?

| Totally agree | Agree | Disagree | Totally disagree | Do not know |
| --- | --- | --- | --- | --- |
|  |  |  |  |  |

1. I feel confident that I collected the urine sample correctly?

| Totally agree | Agree | Disagree | Totally disagree | Do not know |
| --- | --- | --- | --- | --- |
|  |  |  |  |  |

1. Was the user instruction for collecting the urine sample easy to understand?

| Totally agree | Agree | Disagree | Totally disagree | Do not know |
| --- | --- | --- | --- | --- |
|  |  |  |  |  |

**The following question is about your preference for future screening.**

1. If you could choose, what sampling procedure would you prefer?

| □ | Urine collection |
| --- | --- |
| □ | Cervico-vaginal self-sampling |
| □ | GP-based sampling |
| □ | Do not know |
